# Identification of Genomic Regions and the *Isoamylase* Gene for Reduced Grain Chalkiness in Rice

**DOI:** 10.1371/journal.pone.0122013

**Published:** 2015-03-19

**Authors:** Wenqian Sun, Qiaoling Zhou, Yue Yao, Xianjin Qiu, Kun Xie, Sibin Yu

**Affiliations:** 1 National Key Laboratory of Crop Genetic Improvement, Wuhan, China; 2 College of Plant Science and Technology, Huazhong Agricultural University, Wuhan, China; Department of Agriculture and Food Western Australia, AUSTRALIA

## Abstract

Grain chalkiness is an important grain quality related to starch granules in the endosperm. A high percentage of grain chalkiness is a major problem because it diminishes grain quality in rice. Here, we report quantitative trait loci identification for grain chalkiness using high-throughput single nucleotide polymorphism genotyping of a chromosomal segment substitution line population in which each line carried one or a few introduced *japonica* cultivar Nipponbare segments in the genetic background of the *indica* cultivar ZS97. Ten quantitative trait loci regions were commonly identified for the percentage of grain chalkiness and the degree of endosperm chalkiness. The allelic effects at nine of these quantitative trait loci reduced grain chalkiness. Furthermore, a quantitative trait locus (*qPGC8-2*) on chromosome 8 was validated in a chromosomal segment substitution line–derived segregation population, and had a stable effect on chalkiness in a multiple-environment evaluation of the near-isogenic lines. Residing on the *qPGC8-2* region, the isoamylase gene (*ISA1*) was preferentially expressed in the endosperm and revealed some nucleotide polymorphisms between two varieties, Nipponbare and ZS97. Transgenic lines with suppression of *ISA1* by RNA interference produced grains with 20% more chalkiness than the control. The results support that the gene may underlie *qPGC8-2* for grain chalkiness. The multiple-environment trials of the near-isogenic lines also show that combination of the favorable alleles such as the *ISA1* gene for low chalkiness and the *GS3* gene for long grains considerably improved grain quality of ZS97, which proves useful for grain quality improvement in rice breeding programs.

## Introduction

Rice (*Oryza sativa* L.) is one of the most important staple crops, providing food for nearly half of the world’s population. The improvement of grain quality and yield potential is a priority if rice production is to meet the demands of an ever-growing global population. Grain quality encompasses physical appearance and cooking, eating, and nutritional properties. Among these, the key factors determining grain quality are physical appearances related to grain shape and chalkiness. Grain shape or size is characterized by grain length, width, and length-to-width ratio. Chalkiness is usually measured by the percentage of grains with chalkiness (PGC) and the area of opaque parts in the endosperm [1]. High chalkiness is directly associated with inferior cooking and eating qualities, and it generally causes grains to break during the milling process, leading to a decrease in the amount of acceptable and marketable rice. Although rice grain characteristic preferences vary among consumer groups in different countries, slender and translucent kernels without chalkiness are preferred by the majority of rice consumers [2]. Hence, improving rice varieties to have low or no chalkiness is a main objective in rice breeding programs.

Grain chalkiness is a complex trait controlled by multiple genetic factors and influenced by the environment. Numerous quantitative trait loci (QTLs) have been identified for grain chalkiness in various rice mapping populations over the past two decades (http://www.gramene.org). Of these, several major QTLs such as on chromosomes 1, 5, 7, 8, and 9 are stably expressed across multiple environments or various populations [3–7]. Two QTLs for grain chalkiness have been mapped to a small region on chromosomes 7 and 8 [8, 9], respectively. However, the genes responsible for these QTLs have not been determined. Recently, a major QTL for grain chalkiness located on chromosome 5 has been cloned as *Chalk5*, which encodes a vacuolar H+-translocating pyrophosphatase in rice endosperm [10]. The elevated activity of *Chalk5* increases the small vesicle-like structures, coupled with an abnormal decrease in protein body number and size, causing the formation of air spaces among starch granules and resulting in chalky grains.

Grain chalkiness has been reported to be associated with content and structural alteration of starch, which is composed of amylose and amylopectin in endosperm. Amylose is synthesized mainly by ADP-glucose pyrophosphorylase (AGPase) and granule-bound starch synthase (GBSS) in the cereal endosperm, whereas amylopectin biosynthesis is catalyzed by a series of starch biosynthetic isozymes including AGPase, starch synthases (SS), starch branching enzymes (BE), and starch debranching enzymes (DBE) [11]. Plants possess multiple forms of SS (e.g., SSI, SSII, SSIII, SSIV), BE (BEI and BEII), and DBE (isoamylase [ISA], pullulanase [PUL]) that are distinguished based on their substrate specificity [11]. In rice, several mutants deficient in starch-biosynthetic or carbon metabolic genes were reported to have a chalky or floury endosperm phenotype. For example, a mutant of the *OsAGPL2* gene that encodes an AGPase large subunit has a white-core endosperm [12]. A null *SS* mutant caused a deficiency of SSIII, leading to a reduction in the amylopectin content of long chains and in the degree of polymerization [13]. This mutant has a white-core floury endosperm. The mutant of *BEII* produced a white-core endosperm, and the structure of amylopectin and the gelatinization properties of the starch granules were changed [14]. The down-regulation of *BEII* expression led to a severe alteration in starch granule morphology and crystallinity, causing chalky grains [15]. The *ISA* mutants had extremely low or no ISA activity, produced more phytoglycogen or sugary amylopectin in the endosperm, and displayed severe shriveled kernels at maturity [16, 17]. In addition, *pyruvate orthophosphate dikinase* (*PPDKB*) was identified as an important modulator of carbon flow for starch and lipid biosynthesis during grain filling, and its mutant showed a chalky grain phenotype [18]. *Grain Incomplete Filling 1* (*GIF1*) encodes a cell-wall invertase for carbon partitioning during early grain filling. The *gif1* mutant exhibited increased grain chalkiness because of abnormally developed and loosely packed starch granules in the endosperm [19]. The rice *flo6* mutant encoding a protein containing a carbohydrate-binding domain produced small grains with a floury endosperm [20]. These results demonstrate a complex mechanism for chalkiness formation in the rice endosperm. Although many starch-metabolic genes have been characterized in the rice mutants, few corresponding to the QTLs for grain chalkiness have been addressed. Additionally, the majority of mutants have not found applications in rice breeding because of their negative impact on grain quality. Therefore, identification and characterization of desirable QTLs or genes for reduced grain chalkiness are highly required for improvement of grain quality in breeding programs.

In the present study, a chromosomal segment substitution line (CSSL) population derived from *Oryza sativa* spp. *japonica* cv. Nipponbare (NIP) and *indica* cv. Zhenshan 97B (ZS97) was used for detection of grain chalkiness QTLs. ZS97 is an elite variety able to adapt to a wide range of environments. It is widely used as a parent for three-line hybrid rice breeding in China. However, the frequent occurrence of high chalkiness and short grains in ZS97 or its derivative hybrids makes their quality unsatisfactory for consumption. Many efforts have been made to improve the grain quality of ZS97 by transferring favorable genes such as *Waxy* and *GS3* by marker-assisted selection (MAS) [21, 22], but progress in the improvement of the grain chalkiness of ZS97 is still limited because of the lack of desirable QTLs or genes for the trait. Through genome-wide SNP genotyping of the CSSL population, we found several QTLs with the NIP alleles reduced grain chalkiness. One QTL of grain chalkiness on chromosome 8 was validated by using the CSSL-derived population. Furthermore, suppression of *ISA1* that resides on the QTL region caused a reduction of grain chalkiness, indicating that *ISA1* may correspond to the QTL. The substitution lines possessing the favorable alleles that decreased grain chalkiness can be useful for improving grain quality in rice.

## Materials and Methods

Field experiments for evaluation of the following rice materials were conducted at the experimental stations of Huazhong Agricultural University. No permissions were required for these locations/activities. The field studies did not involve endangered or protected species.

### CSSL population and SNP genotyping

A CSSL population consisting of 143 lines was developed using a MAS backcross scheme in which the donor parent was *japonica* cultivar NIP and the recurrent parent was *indica* variety ZS97 [23]. This CSSL population previously genotyped using 175 simple sequence repeat (SSR) markers showed that each line harbors one or more substituted NIP segments of a particular chromosomal region in the genetic background of ZS97 [24]. To determine the genotype more precisely, the CSSLs were re-analyzed using an Infinium RICE6K array (Illumina) containing 5,102 SNPs markers evenly distributed on the 12 rice chromosomes with an average density of 12 SNPs per Mb. The chip hybridization, SNP calling, genotyping, and map construction for the CSSL population were conducted as described [25].

### Bin mapping of QTL

On basis of SNP genotyping, a bin was defined by a unique overlapping substitution segment from the CSSLs as described previously [26]. The chalkiness data for the CSSL population were transformed by arcsin (x)^0.5^, and then normalized by Z-score. QTL analyses of the normalized data with the bins as markers were performed using a linear ridge regression in the R package “ridge” (http://www.r-project.org/), with the ridge parameter chosen automatically [27]. A *t* test for the ridge regression coefficients was conducted for each bin, which was taken as an independent variable in the linear ridge regression model. A significance level of *P* < 0.01 was set as the threshold to declare the presence of a putative QTL in a given bin. If several adjacent bins showed significant *P* values, then the QTL was tentatively located in the most significant bin with the lowest *P* value. The variance explained by each QTL (bin) was decomposed by using lmg from R with the package “relaimpo” [28]. QTL nomenclature followed the principles suggested by McCouch and CGSNL [29].

### Near-isogenic line development and evaluation

A pair of near-isogenic lines (NILs) differing for alleles at the target QTL with a similar genetic background of ZS97 was developed using a marker-assisted backcrossing approach. Briefly, a cross was made between a CSSL possessing the introduced NIP *ISA1* segment and GZ14, an introgression line carrying the grain-size gene *GS3* alleles for long grains in the ZS97 genetic background [30]. Progeny of the cross were backcrossed to ZS97 twice, followed by two rounds of selfing. In each generation, MAS was conducted using the *GS3* and *ISA1* gene-specific markers (S1 Dataset) to develop lines that pyramided the target genes *GS3* and *ISA1*. Two homozygous lines were developed as a pair of NILs and designated as ISA-N and ISA-Z; they had the same long-grain *GS3* but different NIP and ZS97 alleles at *ISA1* in the ZS97 background. During the backcrossing process, one plant heterozygous only at the *ISA1* region was chosen to generate a segregation population for validation of the *ISA1* effect.

The NILs and two parental lines (NIP and ZS97) were evaluated at the Wuhan experimental station during the rice growing seasons for 2 consecutive years. Lines were sown in a nursery bed using sowing dates at regular 18-day intervals, with four sowing dates from May 20 to July 15, 2010, and three sowing dates from May 13 to June 19, 2011. The 25-day-old seedlings were then transplanted in the experimental field in three replicates from each sowing date. Each line was grown in a three-row plot with 10 plants in each row, with a spacing of 16.7 cm between plants and 26.6 cm between rows. In addition, the CSSL population was grown at the experimental field following the same plant spacing conditions used in the growing seasons of 2010 and 2011.

### RNA isolation and expression analysis

Panicles were collected from plants 7 to 10 days after flowering (DAF), immediately frozen in liquid nitrogen, and stored at −80°C until required. RNA was extracted from the panicles using TRIzol Reagent (Invitrogen). cDNA was synthesized using a M-MLV reverse-transcription kit (Invitrogen) according to the manufacturer’s instructions. Semi-quantitative reverse-transcription (RT) and real-time RT polymerase chain reaction (PCR) analysis followed the method described previously [22]. The primers used for RT-PCR are listed in S1 Dataset.

### RNAi construct and transgenic experiment

To construct the RNAi vector for *ISA1*, a 380-bp fragment of the *ISA1* gene was amplified from cDNA of NIP using the primer pair IS1-F and IS1–R (S1 Dataset). The *BamHI–KpnI* and *SacI–SpeI* digested fragments were cloned into the vector pDS1301 under the control of 35S promoter [31]. The RNAi construct was introduced into *Agrobacterium tumefaciens* strain EHA105, and then transferred into the variety NIP using the *Agrobacterium-*mediated transformation method [32].

Transgenic plants containing RNAi constructs were identified by PCR using the primers pMCG1 and pMCG2 (S1 Dataset). Positive T_0_ plants that showed downregulated *ISA* expression were selected to produce T_1_ lines. T_1_ plants that produced a significant quantity of seeds were advanced to the T_2_ generation for progeny testing. The transformed negative was used as a control. The T_2_ families each comprised 21 lines, and 20 to 30 sibling plants per line were grown at the Wuhan experimental site.

### Trait measurements

Mature seeds were harvested, air-dried at room temperature for 3 months, de-husked, and then milled by a grain polisher. Physical appearances such as grain chalkiness, grain length, and grain width of the polished grains were measured using a grain quality analyzer (Model JMWT12, Beijing, China). The PGC and the area of opaque parts in the endosperm were also determined by the grain analyzer. The degree of endosperm chalkiness (DEC) was calculated by these two measured traits. The polished rice was then ground in a mill (FOSS 1093, Cyclotec Sample Mill, Hoganas, Sweden). The pasting properties of ground flour passed through a 100-mesh sieve were measured using a rapid visco analyzer (RVA) (Techmaster, Warriewood, Australia) following the method described by Yan et al. [33]. The RVA profiles included peak viscosity, final viscosity, breakdown viscosity (BDV), setback viscosity (SBV), peak time (PT), and pasting temperature (PAT). All the viscosity parameters were recorded and expressed in centipoise (cP). All measurements were repeated three times. Phenotypic differences among the transgenic lines and the NILs were tested statistically with one-way analysis of variance and Duncan’s test using Statistica [34].

## Results

### QTL regions for grain chalkiness in CSSL

**Fig 1:**
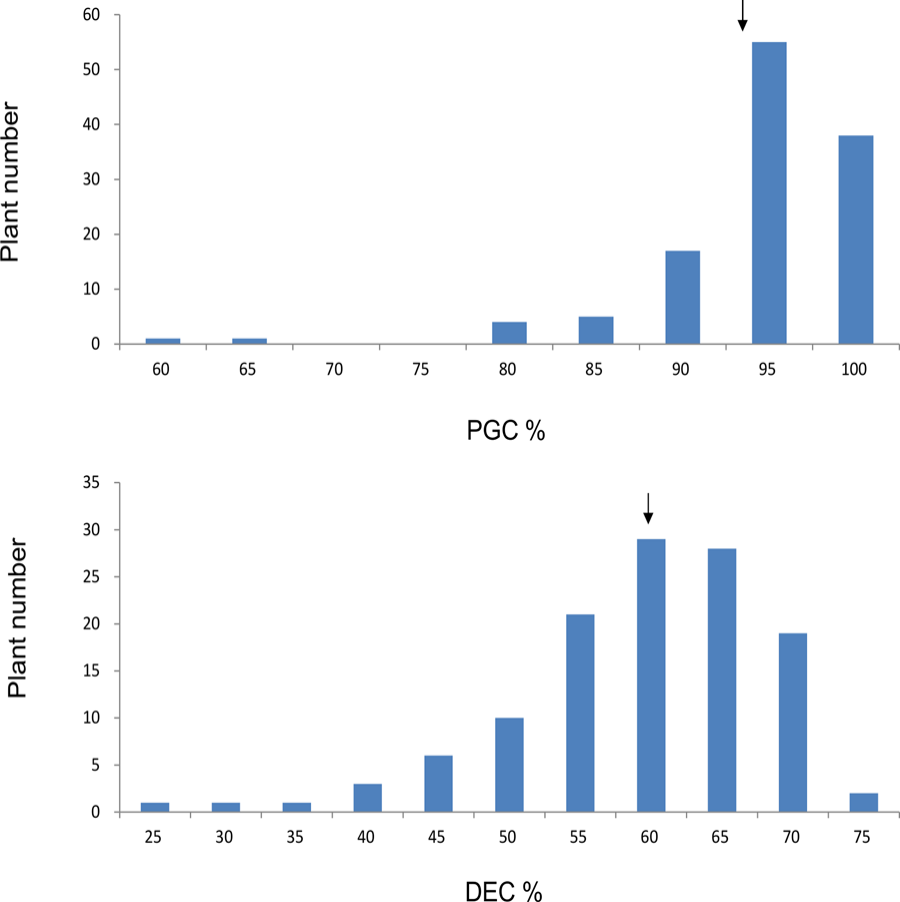
Frequency distribution of grain chalkiness in the CSSL population. (a) The percentage of grain with chalkiness (PGC) and (b) degree of endosperm chalkiness (DEC). The arrows indicate the mean of ZS97.

The two parental lines (NIP and ZS97) differed markedly in PGC and DEC (S2 Dataset). ZS97 had high PGC (93.5%) and DEC (46.1%), which were significantly different from that of NIP (6.1% and 2.2%, respectively). Although the CSSL population comprising 143 lines varied widely in PGC and DEC, most lines had grain chalkiness similar to ZS97, whereas several had significantly lower PGC and DEC than ZS97 (Fig. 1).

The SNP genotyping indicated that the 143 CSSL carried 259 substitution chromosome segments, and each CSSL contained between one and six substituted segments from the NIP in the genetic background of ZS97. A total of 518 bins (defined as B1 to B518) across the whole genome were obtained from the 143 CSSL using the SNP genotyping (S3 Dataset). The physical lengths of the bins ranged from 4.6 Kb to 9.5 Mb, with a median size of 400 Kb.

**Fig 2:**
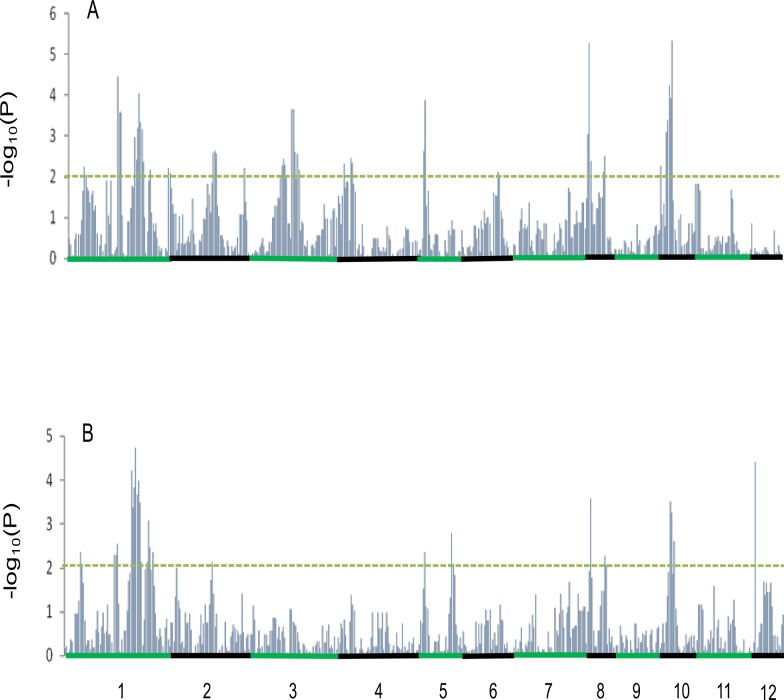
Bin mapping of grain chalkiness QTLs. The bin effects on PGC (a) and DEC (b) along the whole genome in the NIP/ZS97 CSSL population. The green and black bars on the x-axis represent the chromosomes. The -log scale of *P* values are plotted on the y-axis. The dotted lines indicate the threshold with *P* = 0.01.

**Fig 3:**
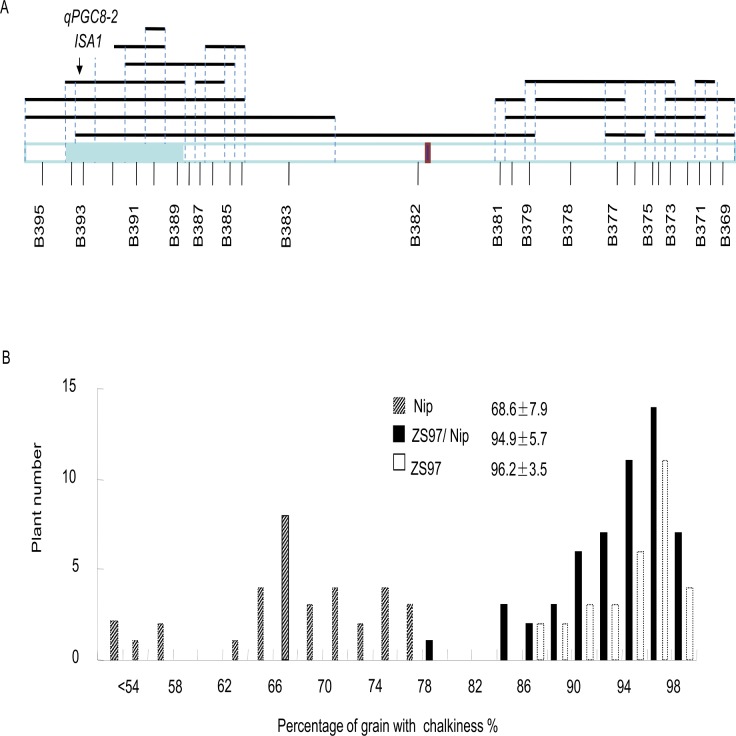
Validation of *qPGC8–2*. (a) The schematic bins on chromosome 8 defined by SNP genotyping of the CSSL population, where one bin contains an introduced *qPGC8–2* (*ISA1*). (b) Grain chalkiness in the three genotypic classes in a CSSL-derived segregation population. Means and standard deviations of grain chalkiness for the three genotypes by the gene marker DE2 are given inside the panel.

QTL analysis of the CSSL population with 518 bin markers identified 17 and 12 QTLs for PGC and DEC, respectively. The detail information on location and effect of the identified QTLs is presented in S3 Dataset. Ten common QTL regions were detected for both PGC and DEC and distributed on chromosomes 1, 2, 5, 8, and 10 (Fig. 2, Table 1). All of these QTLs but one (Bin 109) had the same direction of allelic effect that reduced grain chalkiness. The QTL *qPGC3–1* located on Bin 167 (26.8–28.9 Mb) of chromosome 3 showed the strongest effect on PGC, explaining 7.4% of the variation. The QTL with the largest effect on DEC was mapped to Bin 441 on chromosome 10. The *qPGC8–2* on Bin 393 (24.7–26.1 Mb) of chromosome 8 showed a medium effect on PGC, explaining 3.3% of the variation (Fig. 3a). Notably, using the bin mapping, *qPGC1–2* was located in a 500-kb region containing a starch biosynthesis gene *SSIV*. A large-effect QTL *qPGC5–1* was mapped to Bin 263 of a 1.5-Mb region containing *Chalk5*, a recently cloned rice gene with a significant role in chalkiness formation.

**Table 1:** The common QTLs identified for both PGC and DEC in the ZS/NIP CSSL population using the SNP bin markers.

Chr.	Bin No.	Bin size (Mb)^a^	QTL	PGC			DEC			
				Effect	P value^b^	PVE %	Effect	P value	PVE%	gene^c^
1	B12	6.1–7.1	*qPGC1–1*	-0.135	4.43E-03	4.7	-0.045	5.85E-03	4.9	
1	B39	28.4–28.8	*qPGC1–2*	-0.140	2.93E-03	4.9	-0.070	3.56E-05	3.8	*SSIV*
1	B54	38.6–38.7	*qPGC1–3*	-0.153	1.89E-05	4.5	-0.055	9.13E-05	6.6	
1	B62	40.0–40.2	*qPGC1–4*	-0.125	8.51E-04	1.7	-0.038	6.80E-03	3.4	
2	B109	22.1–23.1	*qPGC2–2*	0.091	7.30E-03	2.9	0.042	2.43E-03	4.9	
5	B263	2.3–3.8	*qPGC5–1*	-0.140	4.51E-03	6.7	-0.063	1.36E-04	4.3	*Chalk5*
8	B382	9.6–15.8	*qPGC8–1*	-0.147	1.68E-03	7.1	-0.071	5.49E-06	4.8	
8	B393	24.6–26.1	*qPGC8–2*	-0.162	2.67E-04	3.3	-0.045	3.17E-03	3.5	*ISA1*
10	B441	11.7–13.2	*qPGC10–2*	-0.129	5.60E-03	5.0	-0.072	4.80E-06	7.4	

^a^ The physical positions of bin size presented according to rice TIGR6.1.

^b^ When two or more consecutive bins were significant, the lowest value is indicated.

^c^ refers to those genes nearby 500 kb of the most significantly associated SNP.

### Validation of *qPGC8–2* and candidate gene analysis

The CSSL line (HJ104) carried a unique NIP substitution segment spanning a 1.4-Mb region (Bin 393) containing *qPGC8–2* (Fig. 3a). It had an average PGC of 72%, significantly lower than that of ZS97, whereas the grain length and width were similar to ZS97 (S2 Dataset). To confirm the effects on chalkiness from *qPGC8–2*, a segregation population composed of 118 individuals derived from the QTL-containing CSSL was evaluated for grain chalkiness and genotyped using the gene-specific marker DE-2. The results showed that plants homozygous and heterozygous for ZS97 alleles at the DE-2 locus had higher grain chalkiness, with an average PGC of 96.2% and 94.9%, respectively (Fig. 3b); both were significantly larger than that of homozygous NIP alleles (68.6%). The results confirmed that existence of *qPGC8–2* with NIP alleles decreased grain chalkiness in a recessive manner.

**Fig 4:**
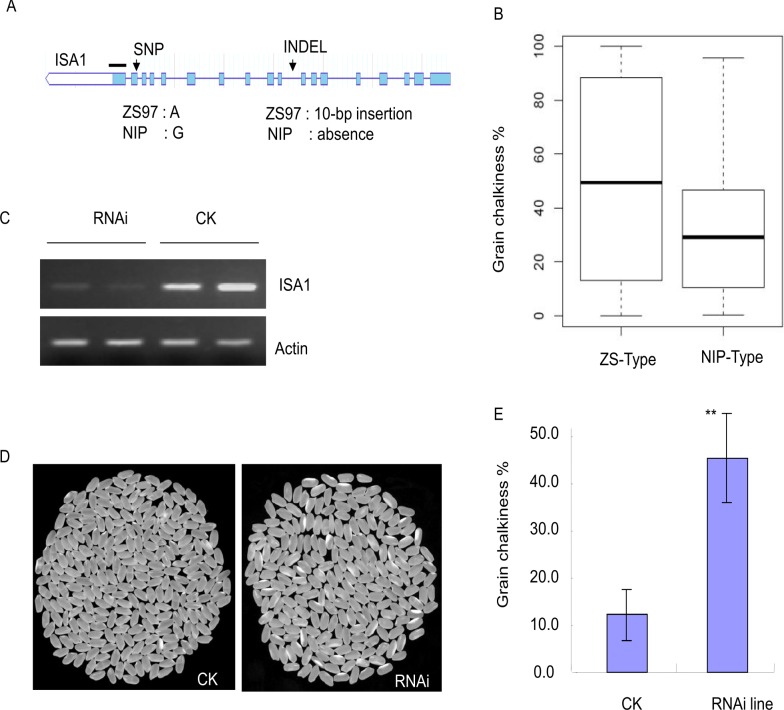
Suppression of *ISA1* causes chalky grains. (a) The gene *ISA1* model showing INDEL and SNP differences between ZS97 and NIP; the exons (rectangles) and introns (lines connecting the exons) are illustrated; the RNAi target domain is indicated by a short bold line above the model. (b) Box plot for PGC levels of 31 NIP type and 73 ZS type varieties at the InDel (the middle line indicates the median, the box indicates the range of the 25th to 75th percentiles of the total data, the whiskers indicate the interquartile range). (c) Quantitative RT-PCR showing the repressed *ISA1* expression in the RNAi line, where the *Actin* gene was used as an internal control. The (d) grain appearances and (e) PGC difference in the *ISA1*-RNAi line and the control. Asterisks (**) indicate mean values are significantly different at *P* < 0.01.

The Bin 393 or *qPGC8–2* region possesses a total of 46 annotated genes that are expressed in rice endosperm. Among these, there are 10 genes involved in carbohydrate metabolism and potentially associated with the QTL (http://rice.plantbiology.msu.edu/). Expression analysis of these 10 genes in various tissues of ZS97 showed two genes (*Os08g40930*, *Os08g41100*) highly expressed in the endosperm at 7 to 10 DAF (S4 Dataset, http://crep.ncpgr.cn/crep-cgi/query_by_tree.cgi). However, only the gene (*Os08g40930*) designated as *ISA1* that encodes isoamylase is preferentially expressed throughout endosperm development, whereas another gene is widely expressed in all assayed tissues of rice (S4 Dataset). Furthermore, sequence comparison of *ISA1* between ZS97 and NIP revealed a 10-bp insertion/deletion (INDEL) in the eighth intron and a nonsynonymous A/G (SNP) in the 17th exon of the gene (Fig. 4a). The nonsynonymous polymorphism was predicted to cause an amino acid change of Thr in ZS97 to Arg in NIP.

To investigate whether these polymorphisms are associated with the grain chalkiness variation, a panel of rice germplasm comprising 73 *indica* and 31 *japonica* varieties was genotyped with the INDEL using the DE-2 marker (S5 Dataset). The results indicate that the samples were classified in two groups, *japonica* type (or NIP type) and *indica* type (or ZS type), and that PGC and DEC of the ZS type are significantly higher than those of the NIP type (*t* test; *P* = 0.008 and *P* = 0.02, respectively; Fig. 4b), suggesting that *ISA1* may be the most likely gene underlying *qPGC8–2*.

### RNAi-mediated suppression of *ISA1* causes chalky grains

To test the possible role of *ISA1* for grain chalkiness, *ISA1-*RNAi transgenic lines were generated by using an RNAi approach. Fifty T_0_ independent transformants were verified positive for *ISA1-*RNAi by PCR. Six of which with moderately suppressed transcription of *ISA1* were allowed to generate T_1_ progeny. These six T_1_ lines and one transgenic-negative line with unchanged expression used as a control were grown at the experimental site, and seeds harvested from every plant were examined for grain chalkiness. All *ISA1-*RNAi lines showed similar heading dates, normal growth, and normal seed shape, but displayed significantly increased chalky grains as compared with the negative control. The average PGC of the RNAi lines ranged from 15.4% to 25.4%, whereas the negative control and wild-type NIP had average PGC of 6.8% and 6.1%, respectively.

The T_2_ RNAi line (NR4) had more grains with white-belly endosperm (Fig. 4c and 4d) and a higher average PGC (42.0%); such higher PGC was also observed in two other RNAi lines (NR2 and NR5) that ranged from 29.0% to 31.5% (S6 Dataset). In contrast, the control line had an average PGC of 12%. The *ISA1-*RNAi with suppressed *ISA1* also affects physicochemical properties of rice grains. In comparison with the control, the RNAi lines had a slightly lower level of BDV and higher SBV, PT, and PAT. In particular, the RNAi line NR4 with a four-fold suppression of ISA1 expression level had significantly decreased BDV (by 50%) and increased SBV (by nearly 150%) relative to the control. However, the amylose content, protein content, and grain size in the RNAi line were not significantly different from those of the control (S6 Dataset). These results indicate that *ISA1* is responsible for the occurrence of grain chalkiness.

### Repressed *ISA1* affects the expression of other starch genes

**Fig 5:**
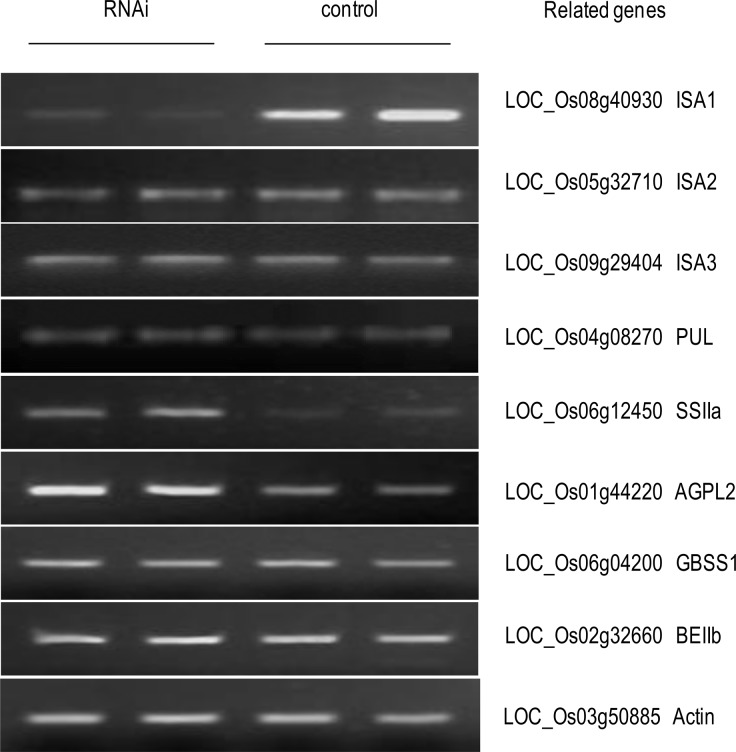
Suppressed *ISA1* influences the expressions of starch synthesis related genes in the rice panicle. Differential expressions of eight starch-related genes between the RNAi line and the control, with the identification numbers based on the rice genome database (http://www.plantbiology.msu.edu); the *Actin* gene was used as an internal control for the semi-quantitative RT-PCR.

To investigate whether downregulation of *ISA1* influences the expression of other starch biosynthesis-related genes, three DBE genes (*ISA* and *PUL*) and four key genes (*AGPL2*, *SSII*, *GBSS1*, and *BEIIb*) preferentially expressed in the endosperm during the grain-filling stage were examined (Fig. 5). As compared with the control, the *ISA1-*RNAi line upregulated transcripts of *AGPL2* and *SSII* but did not alter the expressions of the other five starch-related genes (*ISA2*, *ISA3*, *PUL*, *GBSS1*, and *BEIIb*).

### Stable effect of *qPGC8–2* over multiple rice-growing seasons

**Fig 6:**
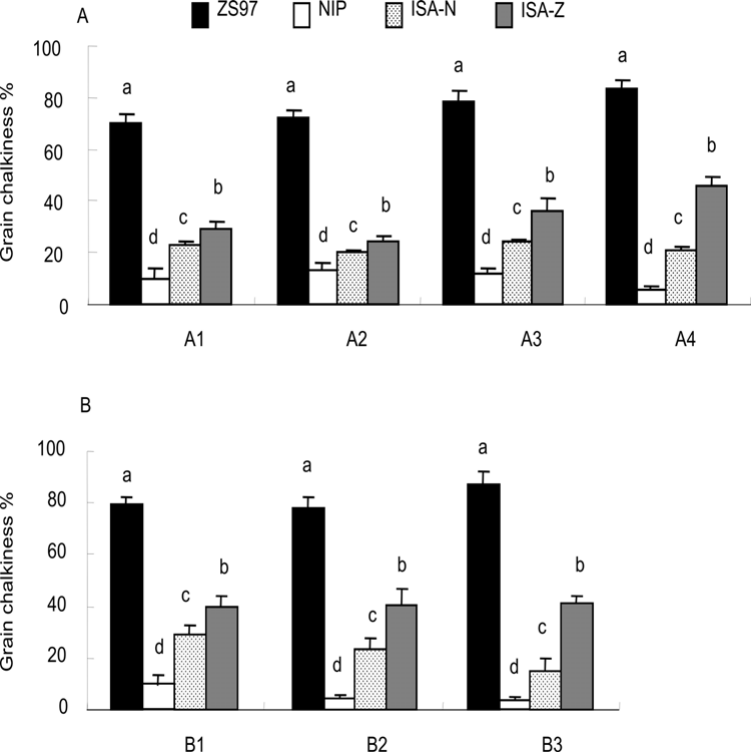
Evaluation of the NILs with contrasting *ISA1* alleles. (a) The trial with four sowing dates (A1–A4) in 2010. (b) The trial with three sowing dates (B1–B3) in 2011. ISA-N and ISA-Z represent the *ISA1* alleles from NIP and ZS97, respectively. Different letters (a, b, c, and d) above the bars indicate significant differences at *P* < 0.01 using Duncan’s test.

To further characterize the genetic effect of *qPGC8–2* on grain chalkiness and its potential application in grain quality, two near-isogenic lines, ISA-N and ISA-Z carrying NIP and ZS97 alleles at *qPGC8–2*, respectively, were evaluated during different growing seasons. The multi-environmental trial indicates that a large variation in PGC observed (from a minimum of 2.2% in NIP to a maximum of 87.3% in ZS97) across different sowing dates, and significant differences in grain chalkiness among the parents and NILs were stable across the environments (Fig. 6). Relative to ZS97, both ISA-N and ISA-Z exhibited longer grain length and significantly lower grain chalkiness. Moreover, a significant difference in chalkiness was also observed between the two NILs: ISA-N had 17% lower PGC than ISA-Z. This is in agreement with the effect of the *qPGC8–2* segment introduced in the CSSL (HJ104), which had approximately 18% less chalkiness than ZS97. In addition, ISA-Z had grains with less chalkiness than ZS97, indicating that the long-grain *GS3* gene introduced may contribute to reduced chalkiness in ZS97.

## Discussion

The present study using the NIP/ZS CSSL population with SNP array identified at least 10 QTLs controlling grain chalkiness; among these, three loci were mapped in regions where the starch-related genes were located (Table 1). For instance, *qPGC1–2* was located in a 500-kb region containing a starch biosynthesis gene *SSIV* on chromosome 1. A large-effect QTL *qPGC5–1* on chromosome 5 was mapped to Bin 263 of a 1.5-Mb region that contains the cloned gene *Chalk5* [10]. *qPGC8–2* in Bin 393 holds the starch-debranching enzyme gene *ISA1*. Notably, *qPGC8–2*, with a stable effect on PGC and DEC, is particularly interesting because it partially overlaps with a QTL region of grain chalkiness that was previously detected in numerous studies across different genetic populations [3–7, 35]. Expression and sequence analyses showed that the starch-related gene *ISA1* residing in the *qPGC-8* region is the most likely candidate gene for the QTL, mainly because of its preferential expression in endosperm and nucleotide polymorphisms associated with the chalkiness variation in large rice samples (S4 Dataset, Fig. 4). Moreover, transgenic analysis showed that the RNAi line with downregulation of ISA1 expression resulted in 20% higher amounts of chalkiness in endosperm, but other agronomic traits were unchanged as compared with the control (Table 1, Fig. 4). In consistent with this observation, the NIP alleles at the *qPGC-8* region introduced into the *indica* ZS97 stably reduced chalky grains approximately 18%, indicating that *ISA1* alleles from NIP and ZS97 may have a different function regarding grain chalkiness.

In addition, the present study demonstrates that the moderate downregulation of *ISA1* expression by RNAi caused an alteration of endosperm physicochemical properties such as breakdown and setback viscosities. The observation is similar to previous reports of the *ISA1* knockdown lines with some physicochemical property changes in starch of barley and rice grains, which may reflect the fine structure of amylopectin and the starch thermal properties [36–39]. Unlike the *ISA1* knockout lines, our RNAi lines had normal amylose content and seed shape. It is of note that grain chalkiness is influenced by the environment, especially the temperature during the grain-filling period [40]. Interestingly, the *ISA1* expression was unaffected during grain-filling of rice at different temperatures, whereas other carbohydrate enzymes preferentially expressed in the endosperm exhibited a liable alteration under high temperatures [41]. The expression pattern of *ISA1* unaltered by the temperature is in line with the stable effect of *qPGC-8*, providing additional support to the idea that *ISA1* may underlie *qPGC8–2* and have a specific role in chalkiness formation in rice grains.

The starch-related genes (*AGPL2*, *GBSSI*, *SSIIa*, *BEIIb*, *ISA1*, and *PUL*) preferentially expressed in the endosperm play essential roles in starch synthesis at the middle stage of seed development [42]. In the present study, suppression of *ISA1* in the RNAi lines upregulated the transcript level of both *AGPL2* and *SSIIa*, but it did not alter that of *GBSSI*, *BEIIb*, and *PUL* (Fig. 5), which is in agreement with the observations of some physicochemical properties that changed. However, accumulation of *PUL* and *BEIIb* transcripts has been observed in an *ISA*-deficient mutant in a previous study [37]. This contrast of results may be a consequence of different levels in ISA expression or activity; this would lead to different amounts of ISA homo-oligomers and selectively affect the expression of other genes in the starch biosynthesis pathway [17, 36]. In addition, the absence of *BEII* in the floury endosperm mutation (*flo2*) of rice led to a downregulation of *GBSSI*, *SSIIa*, and *ISA1* [43]. These results suggest the possible interaction and feedback mechanism between starch-biosynthetic genes, although the mechanisms remain unclear.

Chalkiness and grain shape are the two key physical appearance traits. Long grain and low chalkiness are important breeding goals for rice quality, particularly in *indica* rice, which is widely cultivated in Asia. It is generally considered that grain shape greatly affects endosperm chalkiness and other quality traits such as milling quality in rice. It has been noted that the unfavorable pleiotropic effect of grain size on chalkiness occurs widely in rice and needs to be considered in the efforts to improve rice quality. For example, *GW2* maps to a major QTL for grain width and weight; the increased grain size of *GW2* alleles has a negative effect on grain chalkiness [44, 45]. Our study found that the NIP alleles at *ISA1* stably reduced the grain chalkiness of ZS97 but did not affect the grain length and 1000-grain weight (Fig. 6, S2 Dataset). We also observed a significant difference in chalkiness between the *indica* type and *japonica* type alleles in a wide range of germplasm assessed by the gene-specific marker DE-2 (Fig. 4). These findings provide a breeding strategy in which the *japonica ISA1* as a beneficial allele could be of practical use for improving grain chalkiness in *indica* rice by MAS, although it does not contribute to grain length. In this regard, it is still required for exploring other specific alleles to improve the grain shape. *GS3* is a major QTL for grain length, and the loss-of-function alleles result in the formation of long-grain rice [46]; however, whether this gene influences chalky appearance is undetermined. The present study shows that developed NIL containing the long-grain *GS3* alleles not only significantly increases grain length but also decreases chalkiness in ZS97 (Fig. 6). Therefore, combining the genes such as *ISA1* for low chalkiness and *GS3* for long grains can be used to enhance the quality of *indica* rice such as ZS97.

In conclusion, several QTLs for grain chalkiness were identified in a CSSL population by SNP genotyping. The candidate gene analysis for *qPGC8–2* indicates that *ISA1* may have an underlying role in QTLs for grain chalkiness formation. The present study demonstrates that using the CSSL with high-throughput SNP genotyping is an effective tool for mapping QTLs and can lead to candidate gene(s) for the QTLs of interest. In addition, the other novel large-effect QTLs (e.g., *qPGC8–1* and *qPGC10–2*) may be feasible targets for fine mapping using the CSSL-derived population because they do not co-locate with any starch-biosynthetic genes, and they may offer an excellent opportunity to delve deeper into the genetic basis of grain chalkiness formation. From a breeding perspective, detection of favorable alleles such as *japonica*-type *ISA1* in rice germplasm and development of the gene-specific markers will greatly facilitate precise replacement of alleles in poor-quality varieties using MAS. The available NIL containing the desirable *ISA1* and *GS3* alleles for grain quality, together with the promising lines containing the high-yielding alleles in the common background of ZS97, will serve as excellent parental lines in hybrid rice-breeding programs.

## Supporting Information

S1 Dataset The primers used in this study. (XLS)

S2 Dataset Comparison of grain traits between ZS97 and HJ104 containing *qPGC8–2*. (DOC)

S3 Dataset Chromosomal location and size of each bin, and the QTL regions associated with PGE and DEC from the 143 CSSL genotyped with SNP array. (XLS)

S4 Dataset The expression pattern of several starch-metabolic genes residing in the *qPGC8–2* region. (XLS)

S5 Dataset The rice varieties used in association analysis of grain chalkiness with the INDEL variation in ISA1. (XLS)

S6 Dataset Properties of grain quality for the RNAi lines and the control. (DOC)
