# Supplementary material for: Identification of Genomic Regions and the Isoamylase Gene for Reduced Grain Chalkiness in Rice
Source: PLoS One. 2015 Mar 19;10(3):e0122013. doi: 10.1371/journal.pone.0122013 (PMC4366167; doi:10.1371/journal.pone.0122013)
Supplement: S2 Dataset — (DOC) [file pone.0122013.s002.doc]

**S2-Table S1. Comparison of grain traits between ZS97 and HJ104 containing *qPGC8-2***

| Properties | PGC (%) | AC (%) | Protein(mg/g) | GL (mm) | GW (mm) | KGW (g) |
| --- | --- | --- | --- | --- | --- | --- |
| NIP | 2.6±1.2 | 18.9±0.3 | 118.1±1.1 | 7.64±0.1 | 2.97±0.0 | 23.2±0.3 |
| ZS97 | 90.0±1.0 | 26.1±0.4 | 121.4±0.3 | 7.7±0.0 | 2.2±0.1 | 23.3±0.2 |
| HJ104 | 72.0±2.0 | 25.5±0.6 | 122.2±0.5 | 7.7±0.1 | 2.4±0.1 | 23.4±0.1 |

PGC, percentage of grain with chalkiness; AC, amylose content; GL, grain length; GW, grain width; KGW, 1000-grain weight.
