# Supplementary material for: Identification of Genomic Regions and the Isoamylase Gene for Reduced Grain Chalkiness in Rice
Source: PLoS One. 2015 Mar 19;10(3):e0122013. doi: 10.1371/journal.pone.0122013 (PMC4366167; doi:10.1371/journal.pone.0122013)
Supplement: S6 Dataset — (DOC) [file pone.0122013.s006.doc]

**S6-Table S2. Properties of grain quality for three positive transgenic lines and the control**

| Properties ^a^ | NR2 | NR4 | NR5 | NR7(CK) | NIP (WT) |
| --- | --- | --- | --- | --- | --- |
| PGC (%) | 29.8±9.4** | 42.0±10.4** | 31.5±7.9** | 11.9±4.5 | 2.6±1.2 |
| AC (%) | 19.2±0.8 | 19.1±0.6 | 18.6±0.5 | 19.1±0.5 | 18.9±0.3 |
| Protein (mg/g) | 110.1±1.8 | 111.7±1.2 | 118.6±3.8 | 116.1±1.1 | 118.1±1.1 |
| PV(cp) | 2266.7±374.2 | 2308.9±149.8 | 2484.0±152.0 | 2531.0±113.0 | 2611.0±94.8 |
| FV (cp) | 2728.7±231.3 | 2910.0±171.5 | 2758.0±107.6 | 2780.3±91.2 | 2748.5±98.3 |
| BDV (cp) | 852.7±251.0 | 741.0±25.7** | 1026.0±60.5 | 1103.7±61.0 | 1122.0±93.3 |
| SBV(cp) | 462.0±165.9 | 601.7±38.1** | 274.0±45.5 | 249.3±38.0 | 137.5±3.5 |
| PT (min) | 6.1±0.1 | 6.3±0.1** | 6.1±0.0 | 5.9±0.1 | 6.1±0.0 |
| PAT (℃) | 82.5±9.1 | 87.5±0.4** | 77.5±8.9 | 76.6±7.1 | 74.5±0.1 |
| GL (mm) | 7.82±0.1 | 7.82±0.11 | 7.64±0.1 | 7.74±0.1 | 7.64±0.1 |
| GW (mm) | 2.99±0.0 | 3.01±0.1 | 2.94±0.0 | 2.92±0.1 | 2.97±0.0 |
| KGW (g) | 22.95±0.9 | 22.67±0.5 | 21.86±0.5 | 22.83±0.5 | 23.2±0.3 |

^a^ PGC, percentage of grain with chalkiness; AC, amylose content; PV, peak viscosity; FV, final viscosity; BDV, breakdown viscosity; SBV, setback viscosity; PT, peak time; PAT, pasting temperature; GL, grain length; GW, grain width; KGW, 1000-grain weight. **, significant at *P* < 0.01 against CK by *t*-test.
